# Supplementary material for: The deep-subsurface sulfate reducer Desulfotomaculum kuznetsovii employs two methanol-degrading pathways
Source: Nat Commun. 2018 Jan 16;9:239. doi: 10.1038/s41467-017-02518-9 (PMC5770442; doi:10.1038/s41467-017-02518-9)
Supplement: Supplementary file 1 — Supplementary Information [file 41467_2017_2518_MOESM1_ESM.pdf]

Supplementary information

The deep-subsurface sulfate reducer *Desulfotomaculum kuznetsovii* employs two methanol-degrading pathways

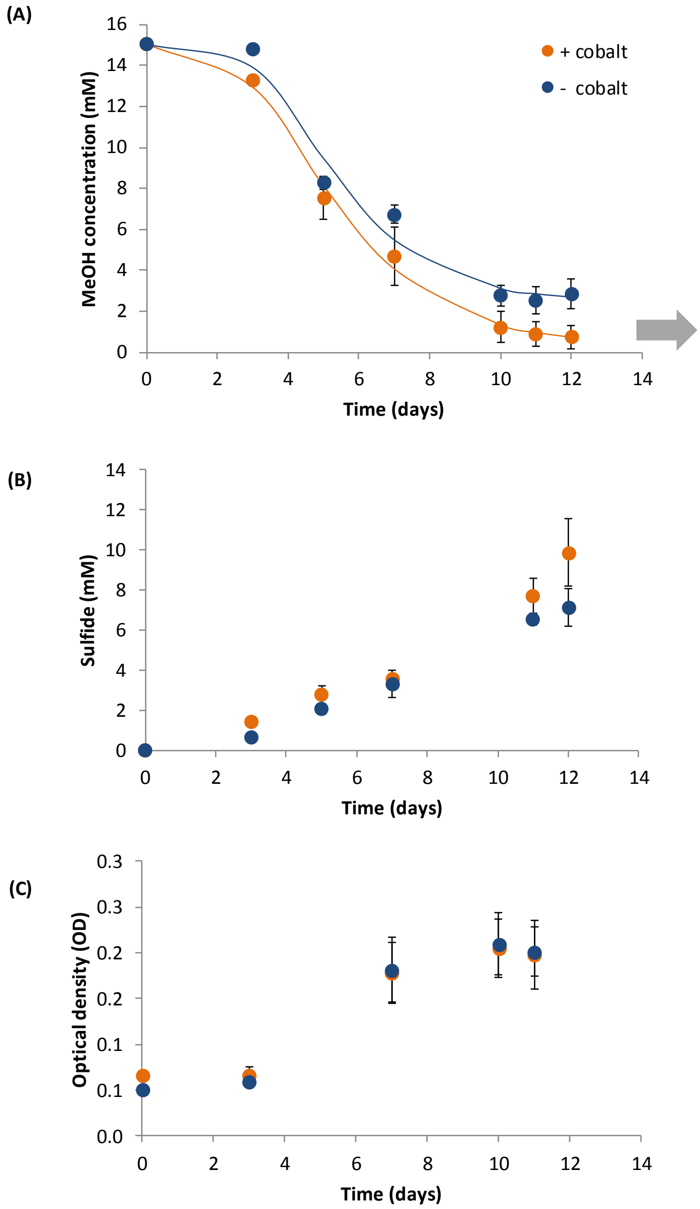

	+ cobalt	- cobalt
methanol conversion rate (mmol L^{-1} day $^{-1}$)	2.63±0.49	2.53±0.06
residual methanol concentration (mM)	0.82±0.53	2.68±0.65

Supplementary Figure 1: (A) methanol utilization, (B) sulfide concentration, and (C) optical density (measured at 600 nm measured in cultures of *D. kuznetsovii* growing with (orange circles) and without (blue circles) cobalt and vitamin B12. Symbols correspond to experimental data with respective standard deviation (*n=3* independent biological replicates).

The lines in (A) represent the predicted data by a modified Gompertz equation[1], used to calculate methanol conversion rate and residual methanol concentration:

$$M(t) = M_0 - M_{max} \exp\left[-exp\left[\frac{R_m.e}{M_{max}}(\lambda - t) + 1\right]\right]$$

where, $M(t)$ is the predicted methanol concentration over time (mM), M_0 is the experimental methanol concentration at t_0 (mM), M_{max} is the predicted methanol concentration at t_0 or during lag phase (mM), R_m is the methanol conversion rate (mmol L^{-1} day^{-1}), *e* equals 2.7182818, and λ is the lag-phase (days).

[1]Zwietering, M.H., Jongenburger, I., Rombouts, F.M., van't Riet, K. Modeling of the bacterial growth curve. Appl. Environm. Microbiol. 56, 1875-1881 (1990).

(A)

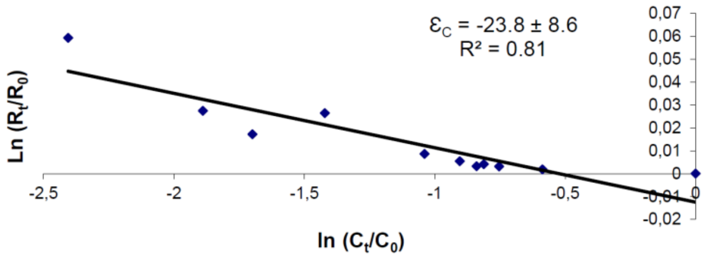

(B)

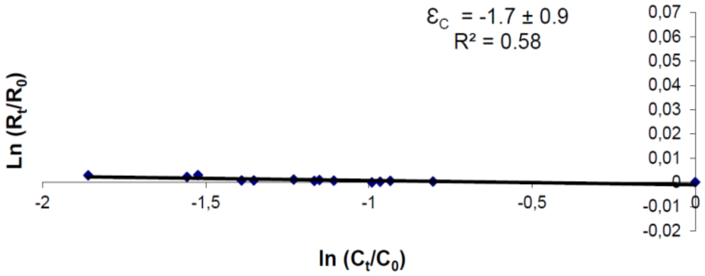

Supplementary Figure 2: Double logarithmic plot according to the Rayleigh equation of the change in carbon isotopic composition $ln(R_t/R_0)$ versus the fraction of methanol $ln(C_t/C_0)$. The lines correspond to a linear regression model and the slope of the curves represent the carbon isotope enrichment factor ε_C. (A) Results for samples taken from cultures cultivated with cobalt and vitamin B12, and (B) results from samples cultivated without cobalt and vitamin B12.
