# Supplementary material for: The deep-subsurface sulfate reducer Desulfotomaculum kuznetsovii employs two methanol-degrading pathways
Source: Nat Commun. 2018 Jan 16;9:239. doi: 10.1038/s41467-017-02518-9 (PMC5770442; doi:10.1038/s41467-017-02518-9)
Supplement: Supplementary file 3 — Description of Additional Supplementary Files [file 41467_2017_2518_MOESM3_ESM.pdf]

Description of Additional Supplementary Files

File Name: Supplementary Data 1

Description: Proteomic data from D. kuznetsovii grown on different substrates: lactate (20 mM), ethanol (20 mM), methanol (20 mM and 5 mM), and methanol (20 mM and 5 mM) without Co and vitamin B12.
